# Reliability and validity of the adapted Greek version of scoliosis research society – 22 (SRS-22) questionnaire

**DOI:** 10.1186/1748-7161-4-14

**Published:** 2009-07-16

**Authors:** Petros D Antonarakos, Labrini Katranitsa, Lefteris Angelis, Aristofanis Paganas, Errikos M Koen, Evangelos A Christodoulou, Anastasios G Christodoulou

**Affiliations:** 1 General Hospital G. Papanikolaou – 1stOrthopaedic Department, Greece; 2 Department of Informatics, Aristotle University of Thessaloniki, Greece; 3 Health Center of Litochoro, Greece; 4 Klinik für Unfall-, Wiederherstellungs- und Orthopädische Chirurgie, St. Marien-Hospital Mülheim an der Ruhr, Kaiserstr, Deutchland

## Abstract

**Background:**

The SRS-22 is a valid instrument for the assessment of the health related quality of life of patients with Idiopathic scoliosis. The SRS-22 questionnaire was developed in USA and has been widely used in the English speaking countries. Recently it has been translated and validated in many other languages. The purpose of this study is to evaluate the reliability and validity of the adapted Greek version of the refined Scoliosis Research Society-22 Questionnaire.

**Methods:**

Following the steps of cross – cultural adaptation the adapted Greek version of the SRS-22 questionnaire and a validated Greek version of the SF-36 questionnaire were mailed to 68 patients treated surgically for Idiopathic Scoliosis. 51 out of the 68 patients returned the 1^st ^set of questionnaires, while a second set was emailed to 30 randomly selected patients of the first time responders. 20 out of the 30 patients returned the 2^nd ^set. The mean age at the time of operation was16,2 years and the mean age at the time of evaluation was 21,2 years. Descriptive statistics for content analysis were calculated. Reliability assessment was determined by estimating Cronbach's α and intraclass correlation coefficient (ICC) respectively. Concurrent validity was evaluated by comparing SRS-22 domains with relevant domains in the SF-36 questionnaire using Pearson's Correlation Coefficient (r).

**Results:**

The calculated Cronbach's α of internal consistency for three of the corresponding domains (pain 0.85; mental health 0.87; self image 0.83) were very satisfactory and for two domains (function/activity 0.72 and satisfaction 0.67) were good. The ICC of all domains of SRS-22 questionnaire was high (ICC>0.70), demonstrating very satisfactory or excellent test/retest reproducibility. Considering concurrent validity all correlations were found to be statistically significant at the 0.01 level among related domains and generally demonstrated high correlation coefficient.

**Conclusion:**

The adapted Greek version of the SRS-22 questionnaire is valid and reliable and can be used for the assessment of the outcome of the treatment of the Greek speaking patients with idiopathic scoliosis.

## Background

There is a strong interest of treating physicians in appropriate and valid measures that allow them to evaluate health-related quality of life (HRQL) outcomes associated with their treatments and to establish the efficacy, or lack thereof, of their interventions [1,2]. Idiopathic scoliosis is a type of deformity that usually does not result in death but may interfere with the quality of life [3]. Although it was common practice to present the outcomes of treatment based on the measurements of spinal correction and balance achieved, nowadays, spine surgeons are more often interested in the reflection of their treatment on patients' health related quality of life.

Haher et al developed a simple, practical and disease specific tool for patients with idiopathic scoliosis [4]. The original questionnaire although sound in general concept, was found to have several psychometric shortcomings [5]. These have been addressed, resulting in the SRS-22 questionnaire that has been shown to have high reliability and concurrent validity in adults, discrimination validity and responsiveness to change in adolescents with scoliosis [6-9].

The overall high performance of the evolved questionnaire led to its wider use in the English speaking countries and also to the wider recognition of its usefulness globally. Up to now the SRS-22 questionnaire has been translated and validated in several languages [10-16]. The purpose of this study is to evaluate the reliability and validity of the adapted Greek version of the refined Scoliosis Research Society-22 Questionnaire [9].

## Methods

The cross-cultural adaptation process was carried out as outlined by Beaton et al. and consisted of 2 stages [17]. First 2 certified bilingual, native Greek speakers produced independently two translations of the original SRS-22 questionnaire. None of the translators was aware of the purpose of the study. Then a synthesis of these 2 translations was conducted by an expert committee consisted of 2 spine surgeons, a general practitioner and a physiotherapist with special interest in spinal disorders. Following that stage the primary Greek version of the SRS-22 questionnaire was given to 30 Greek – speaking patients who had no history of spinal disorders and were asked to define how clear and understandable were the contents of each question. Only minor changes needed to be made and afterwards a backward translation was conducted by two independent native English speakers. The final form of the Greek version of the SRS-22 questionnaire was developed by the expert committee, where all translators also participated and a consensus achieved at the final stage of the cross cultural adaptation [see Additional file 1].

The Greek SRS-22 questionnaire and a previously validated Greek version of SF-36 were mailed to 68 patients who had been surgically treated for idiopathic scoliosis in one spine centre and had a minimum follow-up of 2 years. The mean age of the patients at the time they were enrolled in the study was 21,2 years (16–27 years) while the mean age at the time of operation was 16,2 years (13,8–23,6 years). First mailing consisted of a consent form including a description of the study, SRS-22 questionnaire Greek version, SF-36 questionnaire Greek version, and an addressed and stamped return envelope. 51 patients (75%) responded to the first set of questionnaires. 30 out of the 51 patients were randomly selected for a second set of questionnaires. 20 out of the 30 patients (66%) returned their second survey. The average response time between the first and the second mail was thirty (30) days.

For *content analysis *descriptive statistics (mean, standard deviation, minimum, maximum, and quartiles) were calculated in order to determine the distributions, floor, and ceiling effects. *Reliability *assessment of the Greek version of the SRS-22 questionnaire was determined by calculating Cronbach's α and intraclass correlation coefficient (ICC) values respectively (internal consistency and test/retest reproducibility). *Concurrent validity *was evaluated by comparing SRS-22 domains with relevant domains in the SF-36 questionnaire where correlations were made using Pearson's Correlation Coefficient (r). Statistical analysis was carried out using SPSS16 software.

## Results

### Content analysis

For each domain Table 1 shows the distribution of scores for the five SRS-22 and the nine SF-36 domains (the last one contains only one question). For all of the domains of SRS-22 questionnaire and SF-36 questionnaire, except for the role emotional domain of the SF-36 questionnaire, the percentages of patients with floor effect were less than 6%. The satisfaction with management and the self image domains of SRS-22 questionnaire demonstrated a relatively high percentage of ceiling effect, as well as the role emotional, role physical, social functioning, reported health transition and bodily pain domains of the SF-36 questionnaire.

**Table 1:** Descriptive Statistics on Individual Domain Scores

**SRS-22**							
Domain	n	Mean	Std. Deviation	Minimum (Floor)	Maximum (Ceiling)	% with floor effect	% with ceiling effect

Function/activity	51	4.18	.68	1.60	5.00	2.00%	7.80%

Pain	51	4.07	.84	1.20	5.00	2.00%	9.80%

Mental health	51	3.49	.85	1.00	5.00	2.00%	2.00%

Self image/appearance	51	4.07	.84	1.60	5.00	2.00%	13.7%

Satisfaction with management	51	4.17	.96	1.00	5.00	2.00%	37.3%

**SF-36**							

Domain	n	Mean	Std. Deviation	Minimum	Maximum	% with floor effect	% with ceiling effect

Physical functioning	51	75.08	23.44	0	100	2.00%	3.90%

Role physical	51	75.00	32.40	0	100	5.90%	52.90%

Bodily pain	51	75.10	26.32	0	100	3.90%	23.50%

General health	51	70.29	23.01	10	100	2.00%	9.80%

Vitality	51	60.69	24.78	0	100	3.90%	2.00%

Social functioning	51	73.53	29.75	0	100	2.00%	39.20%

Role emotional	51	71.24	36.53	0	100	13.70%	52.90%

Mental health	51	68.00	23.58	0	100	2.00%	3.90%

Reported health transition	51	69.12	22.69	25	100	2.00%	29.40%

For comparison purposes, the SRS-22 domain scores were converted to a scale from 0 to 100, in order to be comparable with the SF-36 scores [6]. The transformed scores were analyzed by calculating the quartiles of their empirical distributions which are presented, along with the quartiles of the SF-36 domains, in Table 2. It was observed that 50% of the patients in role physical and role-emotional domains of SF-36 questionnaire, scored 100, demonstrating weak spread in the distribution of the responses.

**Table 2:** Distribution of the SRS-22 and SF-36 Domain Scores by Quartiles

**SRS-22 Domains**										
		Function/activity	Pain	Mental health	Self image/appearance	Satisfaction with management				

Quartiles	0%	15	5	0	15	0				
	
	25%	75	70	50	65	62.5				
	
	50%	85	80	65	80	87.5				
	
	75%	90	90	80	93.75	100				
	
	100%	100	100	100	100	100				

**SF-36 Domains**										

		physical functioning	role physical	bodily pain	general health	vitality	social function	role emotional	mental health	reported health transition

Quartiles	0%	0	0	0	10	0	0	0	0	25
	
	25%	70	50	67,5	60	45	50	33,33	56	50
	
	50%	80	100	77,5	80	70	87,5	100	72	50
	
	75%	90	100	90	85	80	100	100	88	100
	
	100%	100	100	100	100	100	100	100	100	100

### Reliability

The *internal consistency reliability *scores are shown in Table 3. The calculated Cronbach's α of internal consistency for three of the corresponding domains (pain 0.85; mental health 0.87; self image 0.83) were very satisfactory (within the range 0.80–0.89) and for two domains (function/activity 0.72 and satisfaction 0.67) were good (within the range 0.50–0.79) [10]. The internal consistency for all the domains of the SF-36 questionnaire is also demonstrated in table 3 and showed good to excellent reliability.

**Table 3:** Internal Consistency Reliability (Cronbach's α)

**SRS-22 Domain**	**α**	**SF-36 Domain**	**α**
Function/activity	0.75	Physical functioning	0.85

Pain	0.85	Role physical	0.74

Mental health	0.87	Bodily pain	0.89

Self image/appearance	0.83	General health	0.81

Satisfaction with management	0.67	Vitality	0.86

		Social functioning	0.87
	
		Role emotional	0.72
	
		Mental health	0.90

The *test/retest reproducibility *correlations are shown in Table 4. The ICC of all domains of SRS-22 questionnaire was high (ICC>0.70), demonstrating very satisfactory or excellent test/retest reproducibility. Two of the domains of SF-36 questionnaire demonstrated excellent test-retest (physical functioning and role physical) while the other domains demonstrated lower reproducibility, especially the role emotional.

**Table 4:** Test-Retest Reproducibility as Determined by Intraclass Correlation Coefficient

**SRS-22 Domain**	**ICC**	**SF-36 Domain**	**ICC**
Function/activity	0.93	Physical functioning	0.94

Pain	0.82	Role physical	0.87

Mental health	0.79	Bodily pain	0.62

Self image/appearance	0.83	General health	0.54

Satisfaction with management	0.72	Vitality	0.58
		Social functioning	0.52

		Role emotional	0.18
	
		Mental health	0.57
	
		Reported health transition	0.44

### Concurrent validity

*Concurrent validity *based on the comparison with the SF-36 questionnaire is shown in Table 5. All correlations were found to be statistically significant at the 0.01 level among related domains and generally demonstrated high correlation coefficient. Two domains (pain vs. bodily pain and mental health vs. mental health index) had excellent correlation (r = 0.87 and 0.89 respectively).

**Table 5:** Concurrent validity of SRS-22 Domains with Relevant SF-36 Domains as Determined by Pearson Correlation Coefficient (r)

**SRS-22 Domains**	**SF-36 Domains**	**Pearson r**	**P**
Function/activity	Role physical	0.65**	0.000
	
	Physical functioning	0.79**	0.000
	
	Bodily pain	0.67**	0.000
	
	General health	0.71**	0.000

Pain	Bodily pain	0.,87**	0.000
	
	Role physical	0.62**	0.000
	
	Physical functioning	0.61**	0.000

Self image	General health	0.74**	0.000
	
	Social function	0.59**	0.000
	
	Physical functioning	0.60**	0.000

Mental health	Mental health	0.89**	0.000
	
	Social function	0.71**	0.000
	
	Vitality	0.74**	0.000
	
	Physical functioning	0.64**	0.000

Satisfaction with management	Physical functioning	0.507**	0.000
	
	Role physical	0.387**	0.005
	
	Bodily pain	0.631**	0.000
	
	General health	0.595**.	0.000

**. Correlation is significant at the 0.01 level (2-tailed).

## Discussion

A cultural and linguistic adaptation of the SRS-22r questionnaire was done to evaluate its reliability and validity for the Greek-speaking patients with idiopathic scoliosis. The transcultural adaptation of the SRS-22 from English to Greek presented here was based on the method proposed by Beaton et al [17]. Recent publications of translated versions of the original SRS-22 questionnaire in to Spanish, Turkish, Chinese, Japanese, Polish and French used similar methods to test the validity and reliability [10-16]. We also included an additional pretest of the Greek version to evaluate whether readers without medical professional background would understand the terms which led us to perform minor linguistic adaptations to the primary translation and helped to achieve a final consensus form of the questionnaire [12].

Content analysis has shown similar results with other studies previously published in the literature. Score distribution between best (ceiling) and worst (floor) outcomes is the means by which an instrument can be evaluated to determine its ability to assess the full range of disease severity and is an essential attribute of a questionnaire. The Greek version of the SRS-22 questionnaire has shown a wide range of scores documenting its capacity to respond to a range of patient perceived outcomes. The metric characteristics of the Greek version of the SRS-22r show an evident ceiling effect for the domains of self image and satisfaction with treatment. A tendency toward this effect is also clear in the scoring for function and pain. This would be expected and hoped for in this particular patient population being studied.

The internal consistency of the Greek version is slightly inferior to that of the original questionnaire (0.79 vs 0.86) [6]. Although this tendency has been observed in other transcultural adaptations of questionnaires, the lower internal consistency of function and satisfaction domain was principally responsible for the lower Cronbach's α value [16]. The domains "function" and "satisfaction" are difficult to interpret. Their scores are strongly influenced by many external factors the most important being patients' expectations, level of education and type of profession, financial aspects, spare time and individual level of activity [15]. These and possible other unknown factors can change the scores independently of the quality of the transcultural adaptation. The fact that the other domains showed very satisfactory Cronbach's values confirms the precise measurements of the questionnaire. Regarding the lower internal consistency of the satisfaction domain, it has been also reported by Cheung et al similarly and was attributed to the discrepancy between the patients' expectations and the final cosmetic result [12]. In our case we have included to the study patients with severe forms of idiopathic scoliosis which may have influenced the values of that particular domain. A further study correlating the type and the severity of scoliosis with the adapted Greek version of the SRS-22 questionnaire is currently in progress and may further enlighten the correlation between patients' satisfaction and capabilities of correction achieved [7,8]. In general the internal consistency of the adapted Greek version of the SRS-22 questionnaire has shown one of the best values reported in the literature [10-16].

Test-retest reliability reached very satisfactory reproducibility. The ICC of all domains of SRS-22 questionnaire was high (ICC>0.70), demonstrating very satisfactory or excellent test/retest reproducibility.

Regarding the concurrent validity, all correlations were found to be statistically significant at the 0.01 level among related domains of the SF-36 and generally demonstrated high correlation coefficient. Two domains (pain vs. bodily pain and mental health vs. mental health index) had excellent correlation (r = 0.87 and 0.89 respectively). Regarding the domain "satisfaction with management" that has been presented with relative low correlation values in other studies our results showed values ranging from 0.387 to 0.631, which are lower than the original version but still satisfactory [8,12,15]. Furthermore, Lai et al have shown that there is an intrinsically poor correlation between the "satisfaction with management" domain of SRS-22 questionnaire and the relative domains of the SF-36, which possibly reflects to the current study [18].

## Conclusion

Our statistical results have confirmed that the adapted Greek version of the SRS-22 questionnaire is valid and reliable and covers a wide range of patients' perceived outcomes. Even so the interpretation of the outcomes needs to be made carefully, while further studies need to evaluate the discrimination validity of the adapted questionnaire.

## Competing interests

The authors declare that they have no competing interests.

## Authors' contributions

PA participated to the study design and carried out the data analysis and manuscript drafting. LK carried out aacquisition and interpretation of data. LA carried out analysis and interpretation of data as well as manuscript drafting. AP participated to the design of the study and carried out the stages of transcultural adaptation. EK carried out acquisition of data. EC participated to the design of the study and acquisition of data. AC conceived the study, participated to coordination and carried out the final corrections draft of the manuscript. All authors read and approved the final manuscript.

## Supplementary Material

Additional file 1 the Greek SRS-22 questionnaire. Click here for file

## References

[B1] Swiontkowski MF, Buckwalter JA, Keller RB, Haralson R (1999). Symposium: the outcomes movement in orthopaedic surgery: Where we are and where we should go. J Bone Joint Surg Am.

[B2] Daum WJ, Brinker MR, Nash DB (2000). Quality and outcome determination in health care and orthopaedics: evolution and current structure. J Am Acad Orthop Surg.

[B3] Pehrsson K, Larsson S, Oden A, Nachemson A (1992). Long term follow-up of patients with untreated scoliosis: a study of mortality, causes of death, and symptoms. Spine.

[B4] Haher TR, Gorup JM, Shin TM, Homel P, Merola AA, Grogan DP, Pugh L, Lowe TG, Murray M (1999). Results of the Scoliosis Research Society instrument for evaluation of surgical outcome in adolescent idiopathic scoliosis: a multicenter study of 244 patients. Spine.

[B5] Asher MA, Lai SM, Burton DC (2000). Further development and validation of the Scoliosis Research Society (SRS) Outcomes Instrument. Spine.

[B6] Asher M, Min Lai S, Burton D, Manna B (2003). The reliability and concurrent validity of the Scoliosis Research Society-22 patient questionnaire for idiopathic scoliosis. Spine.

[B7] Asher M, Min Lai S, Burton D, Manna B (2003). Scoliosis Research Society-22 patient questionnaire: responsiveness to change associated with surgical treatment. Spine.

[B8] Asher M, Min Lai S, Burton D, Manna B (2003). Discrimination validity of the Scoliosis Research Society-22 patient questionnaire: relationship to idiopathic scoliosis curve pattern and curve size. Spine.

[B9] Asher MA, Lai SM, Burton DC (2006). Refinement of the SRS-22 health-related quality of life questionnaire function domain. Spine.

[B10] Alanay A, Cil A, Berk H, Acaroglu RE, Yazici M, Akcali O, Kosay C, Genc Y, Surat A (2005). Reliability and validity of adapted Turkish version of Scoliosis Research Society-22 (SRS-22) questionnaire. Spine.

[B11] Bago J, Climent JM, Ey A, Perez-Grueso FJ, Izquierdo E (2004). The Spanish version of SRS-22 patient questionnaire for idiopathic scoliosis. Spine (Phila Pa 1976).

[B12] Cheung KM, Senkoylu A, Alanay A, Genc Y, Lau S, Luk KD (2007). Reliability and concurrent validity of the adapted Chinese version of Scoliosis Research Society-22 (SRS-22) questionnaire. Spine.

[B13] Hashimoto H, Sase T, Arai Y, Maruyama T, Isobe K, Shouno Y (2007). Validation of Japanese Version of the Scoliosis Research Society – 22 patient questionnaire among idiopathic scoliosis patients in Japan. Spine (Phila Pa 1976).

[B14] Glowacki M, Misterska E, Laurentowska M, Mankowski P (2009). Polish adaptation of scoliosis research society-22 questionnaire. Spine.

[B15] Niemeyer T, Schubert C, Halm HF, Herberts T, Leichtle C, Gesicki M (2009). Validity and reliability of an adapted german version of scoliosis research society-22 questionnaire. Spine.

[B16] Beauséjour M, Joncas J, Goulet L, Roy-Beaudry M, Parent S, Grimard G, Forcier M, Lauriault S, Labelle H (2009). Reliability and validity of adapted French Canadian version of Scoliosis Research Society Outcomes Questionnaire (SRS-22) in Quebec. Spine.

[B17] Beaton DE, Bombardier C, Guillemin F (2000). Guidelines for the process of cross-cultural adaptation of self-report measures. Spine.

[B18] Lai SM, Asher M, Burton D (2006). Estimating SRS-22 quality of life measures with SF-36: application in idiopathic scoliosis. Spine.

