# Supplementary material for: Reliability and validity of the adapted Greek version of scoliosis research society – 22 (SRS-22) questionnaire
Source: Scoliosis. 2009 Jul 16;4:14. doi: 10.1186/1748-7161-4-14 (PMC2720382; doi:10.1186/1748-7161-4-14)
Supplement: Additional file 1 — the Greek SRS-22 questionnaire. [file 1748-7161-4-14-S1.doc]

**Additional File 1**

**SRS-22r Ερωτηματολόγιο Ασθενούς**

Ημερομηνία: ____

Όνομα Ασθενούς: __­­­­_______ ____________Ημερομηνία Γέννησης:___________

Ηλικία: ______+______

Έτη Μήνες

**ΟΔΗΓΙΕΣ:** Εκτιμούμε προσεκτικά την κατάσταση της μέσης /πλάτης σας και είναι **ΣΗΜΑΝΤΙΚΟ ΝΑ ΑΠΑΝΤΗΣΕΤΕ ΜΟΝΟΣ ΣΑΣ ΣΕ ΚΑΘΕ ΜΙΑ ΑΠΟ ΤΙΣ ΕΡΩΤΗΣΕΙΣ.** Παρακαλώ **ΣΗΜΕΙΩΣΤΕ με (Χ) ΤΗΝ ΚΑΛΥΤΕΡΗ ΑΠΑΝΤΗΣΗ ΓΙΑ ΚΑΘΕ ΕΡΩΤΗΣΗ.**

1. **Ποιο από τα παρακάτω περιγράφει καλύτερα πόσο πόνο έχετε αισθανθεί κατά τη διάρκεια των τελευταίων 6 μηνών ;**
   - Καθόλου
   - Ήπιος
   - Μέτριος
   - Μέτριος έως σοβαρός
   - Σοβαρός

1. **Ποιο από τα παρακάτω περιγράφει καλύτερα πόσο πόνο έχετε αισθανθεί τον τελευταίο μήνα ;**
   - Καθόλου
   - Ήπιος
   - Μέτριος
   - Μέτριος έως σοβαρός
   - Σοβαρός
2. **Κατά τη διάρκεια των τελευταίων 6 μηνών ήσασταν ένα πολύ νευρικό άτομο ;**
   - Καθόλου
   - Λίγες φορές
   - Κάποιες φορές
   - Τις περισσότερες φορές
   - Συνεχώς
3. **Αν έπρεπε να περάσετε το υπόλοιπο της ζωής με την μέση σας στην κατάσταση που είναι τώρα, πως θα νιώθατε για αυτό ;**

- Πολύ ευχαριστημένος
- Κατά κάποιο τρόπο ευχαριστημένος
- Ούτε ευχαριστημένος ούτε δυσαρεστημένος
- Κατά κάποιο τρόπο δυσαρεστημένος
- Πολύ δυσαρεστημένος

1. **Ποιο είναι το σημερινό επίπεδο δραστηριοτήτων σας ;**

- Κατάκοιτος /η
- Κατά κύριο λόγο καμία δραστηριότητα
- Ελαφρά εργασία και ελαφρά άσκηση
- Μέτρια εργασία και μέτρια άσκηση
- Πλήρεις δραστηριότητες χωρίς περιορισμό

1. **Πως φαίνεστε με ρούχα ;**

- Πολύ καλά
- Καλά
- Μέτρια
- Άσχημα
- Πολύ άσχημα

1. **Τους τελευταίους 6 μήνες είχατε τόσο τις "μαύρες" σας που τίποτα δεν μπορούσε να** **σας φτιάξει τη διάθεση ;**

- Πολύ συχνά
- Συχνά
- Κάποιες φορές
- Σπάνια
- Ποτέ

1. **Αισθάνεστε πόνο στη μέση κατά την ανάπαυση ;**

- Πολύ συχνά
- Συχνά
- Κάποιες φορές
- Σπάνια
- Ποτέ

1. **Ποιο είναι το τωρινό επίπεδο δραστηριότητας σας στη δουλειά /σχολείο ;**

- 100% φυσιολογικό
- 75% φυσιολογικό
- 50% φυσιολογικό
- 25% φυσιολογικό
- 0% φυσιολογικό

1. **Ποιο από τα παρακάτω περιγράφει καλύτερα την εμφάνιση του κορμού σας. Ορίζεται** **ως το ανθρώπινο σώμα εκτός από το κεφάλι και τα άκρα ;**

- Πολύ καλή
- Καλή
- Μέτρια
- Κακή
- Πολύ κακή

1. **Ποιο από τα παρακάτω περιγράφει καλύτερα την χρήση αναλγητικών που κάνετε για την μέση σας ;**

- Καμιά
- Μη ναρκωτικά αναλγητικά μια φορά την εβδομάδα η λιγότερο
- Μη ναρκωτικά αναλγητικά καθημερινά
- Ναρκωτικά αναλγητικά μια φορά την εβδομάδα η λιγότερο
- Ναρκωτικά αναλγητικά καθημερινά

1. **Η μέση σας περιορίζει την ικανότητα σας να κάνετε πράγματα στο σπίτι ;**

- Ποτέ
- Σπάνια
- Κάποιες φορές
- Συχνά
- Πολύ συχνά

1. **Έχετε νιώσει γαλήνιος και ήρεμος τους τελευταίους 6 μήνες;**

- Συνέχεια
- Τις περισσότερες φορές
- Κάποιες φορές
- Λίγες φορές
- Καθόλου

1. **Αισθάνεστε ότι η κατάσταση της μέσης σας επηρεάζει τις προσωπικές σας σχέσεις ;**

- Καθόλου
- Ελάχιστα
- Ήπια
- Μέτρια
- Σοβαρά

1. **Έχετε εσείς και /ή η οικογένεια σας οικονομικές δυσκολίες εξαιτίας της μέσης σας ;**

- Σοβαρές
- Μέτριες
- Ήπιες
- Ελάχιστες
- Καθόλου

1. **Τους τελευταίους 6 μήνες έχετε αισθανθεί άκεφος και μελαγχολικός /η ;**

- Ποτέ
- Σπάνια
- Κάποιες φορές
- Συχνά
- Πολύ συχνά

1. **Τους τελευταίους 3 μήνες έχετε πάρει κάποιες μέρες άδεια από τη δουλειά,** **συμπεριλαμβανομένης της οικιακής εργασίας, ή του σχολείου εξαιτίας του πόνου στη μέση ;**

- 0 ημέρες
- 1 ημέρα
- 2 ημέρες
- 3 ημέρες
- 4 ή περισσότερες ημέρες

1. **Η κατάσταση της μέση σας περιορίζει τις εξόδους σας με φίλους /οικογένεια;**

- Ποτέ
- Σπάνια
- Κάποιες φορές
- Συχνά
- Πολύ συχνά

1. **Αισθάνεστε ελκυστικός /η με την τωρινή κατάσταση της μέσης σας;**

- Ναι, πολύ
- Ναι, κατά κάποιο τρόπο
- Ούτε ελκυστικός /η ούτε μη ελκυστικός /η
- Όχι, όχι πολύ
- Όχι, καθόλου

1. **Είσαστε καθόλου ευτυχισμένοι κατά τη διάρκεια των τελευταίων 6 μηνών ;**

- Καθόλου
- Λίγες φορές
- Κάποιες φορές
- Τις περισσότερες φορές
- Συνέχεια

1. **Είστε ικανοποιημένος /η με τα αποτελέσματα της θεραπείας της μέσης σας;**

- Πολύ ικανοποιημένος /η
- Ικανοποιημένος /η
- Ούτε ικανοποιημένος /η ούτε δυσαρεστημένος /η
- Δυσαρεστημένος /η
- Πολύ δυσαρεστημένος /η

1. **Θα δεχόσασταν ξανά την ίδια θεραπεία αν είχατε την ίδια κατάσταση;**
   - Σίγουρα ναι
   - Μάλλον ναι
   - Δεν είμαι σίγουρος/η
   - Μάλλον όχι
   - Σίγουρα όχι

Σας ευχαριστώ που συμπληρώσατε αυτό το ερωτηματολόγιο. Σας παρακαλώ σχολιάστε αν το επιθυμείτε.
